# What does effective population size tell us about loss of allelic variation?

**DOI:** 10.1111/eva.13733

**Published:** 2024-06-21

**Authors:** Fred W. Allendorf, Ola Hössjer, Nils Ryman

**Affiliations:** ^1^ Division of Biological Sciences University of Montana Missoula Montana USA; ^2^ Department of Mathematics Stockholm University Stockholm Sweden; ^3^ Department of Zoology Stockholm University Stockholm Sweden

**Keywords:** allelic variation, bottleneck, drift‐mutation equilibrium, effective population size, genetic drift, heterozygosity

## Abstract

There are two primary measures of the amount of genetic variation in a population at a locus: heterozygosity and the number of alleles. Effective population size (*N*_e_) provides both an expectation of the amount of heterozygosity in a population at drift‐mutation equilibrium and the rate of loss of heterozygosity because of genetic drift. In contrast, the number of alleles in a population at drift‐mutation equilibrium is a function of both *N*_e_ and census size (*N*_C_). In addition, populations with the same *N*_e_ can lose allelic variation at very different rates. Allelic variation is generally much more sensitive to bottlenecks than heterozygosity. Expressions used to adjust for the effects of violations of the ideal population on *N*_e_ do not provide good predictions of the loss of allelic variation. These effects are much greater for loci with many alleles, which are often important for adaptation. We show that there is a linear relationship between the reduction of *N*_C_ and the corresponding reduction of the expected number of alleles at drift‐mutation equilibrium. This makes it possible to predict the expected effect of a bottleneck on allelic variation. Heterozygosity provides good estimates of the rate of adaptive change in the short‐term, but allelic variation provides important information about long‐term adaptive change. The guideline of long‐term *N*_e_ being greater than 500 is often used as a primary genetic metric for evaluating conservation status. We recommend that this guideline be expanded to take into account allelic variation as well as heterozygosity.

## 1 INTRODUCTION

There are two primary measures of the amount of genetic variation in a population: heterozygosity and the number of alleles. Heterozygosity is generally measured by expected heterozygosity (*H*), which is the frequency of heterozygotes expected at a locus in Hardy–Weinberg proportions.

The inbreeding effective population size (*N*_e_) describes the rate of loss of heterozygosity in a population. Effective population size is one of the most important parameters in evolutionary biology and conservation (Waples, 2022). Effective population size is often used to determine conservation status or to inform management. However, populations with the same *N*_e_, and therefore the same rate of decline of heterozygosity, can experience very different rates of loss of allelic variation (Allendorf, 1986; Ryman et al., 1995). Therefore, we must consider more than just effective population size when considering the rate of loss of genetic variation in populations.

There has been a confusing variety of symbols used in the literature to describe the amount of allelic variation at a locus. We have chosen to use the following symbols (Table 1). We use *A*_n_ to represent the number of alleles present at a locus. We use *A*_R_ to represent allelic richness, which is a measure of the number of alleles present in a sample that takes into account sample size (El Mousadik & Petit, 1996). Finally, we use *A*_D_ to represent allelic diversity, which we define as the proportion of alleles remaining at a locus after a bottleneck of one or more generations (Allendorf, 1986). We have not used the effective number of alleles (*A*_e_) because it provides no more information about the number of alleles present at a locus than does heterozygosity. The effective number of alleles is the number of alleles that if equally frequent would result in the observed heterozygosity (Kimura & Crow, 1964).

**TABLE 1:** Notation used to describe parameters in this paper.

Term	Definition
*A*_D_	Allelic diversity, proportion of alleles remaining at a locus after a bottleneck of one or more generations
*A*_e_	Effective number of alleles at a locus
*A*_n_	The number of alleles at a locus
*A*_R_	Allelic richness, number of alleles at a locus in a sample that takes into account sample size
*H*	Expected frequency of heterozygotes in Hardy–Weinberg proportions
*N*	Population size of an ideal Wright–Fisher population (*N* = *N*_e_ = *N*_C_)
*N*_C_	Census population size
*N*_e_	Inbreeding effective population size
*p*	Allele frequency
*t*	Number of generations
*μ*	Mutation rate

Our objective in this paper is to describe under what conditions effective population size is insufficient to describe the maintenance and loss of allelic variation. That is, under what circumstances is it essential to consider the rate of loss of allelic variation as well as effective population size in considering the conservation of a species or population?

## 2 EQUILIBRIUM

In the absence of natural selection, the amount of genetic variation within a population at equilibrium will be a balance between the gain of variation as a function of the neutral mutation rate (*μ*) and the loss of genetic variation by genetic drift. This is drift‐mutation equilibrium.

At drift‐mutation equilibrium, *H* in a population is a function of *N*_e_ and mutation rate (*μ*). The average expected heterozygosity (*H*) at a locus (or over many loci with the same mutation rate, *μ*) is approximately the following with the infinite allele model of mutation (Kimura & Crow, 1964; Crow & Kimura, 1970, page 323):

(1) $$H\approx \frac{4N_{e}\mu }{\left(4N_{e}\mu +1\right)}$$

**FIGURE 1:**
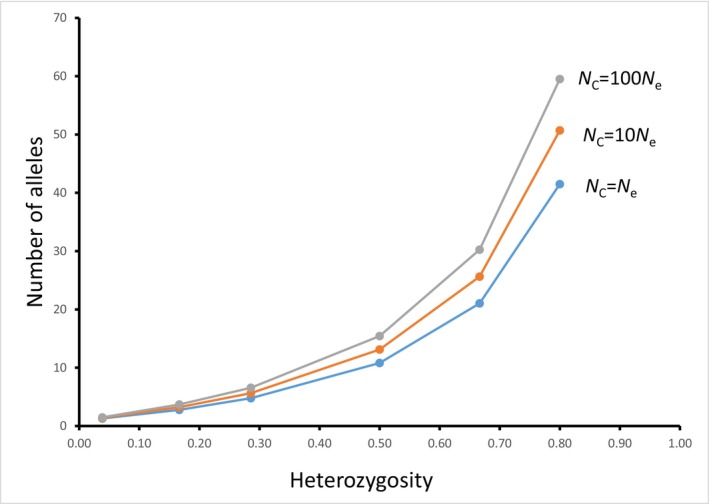
Average number of alleles expected per locus at mutation‐drift equilibrium for different census population sizes (*N*_C_) under the infinite alleles model with a mutation rate of 10^−5^ (Equation 2) and different values of *N*_e_. Heterozygosity is calculated with Equation (1); for example, *H* = 0.500 when *μ* = 10^−5^ and *N*_e_ = 25,000.

In contrast, the expected number of alleles (*A*_n_) at drift‐mutation equilibrium is a function of both effective population size and census population size (*N*_C_), as well as *μ* (Kimura, 1968; Crow & Kimura, 1970, p. 455):

(2) $$E\left(A_{n}\right)=4N_{e}\mu \int_{\frac{1}{2N_{C}}}^{1}{\left(1-p\right)}^{4N_{e}\mu -1}p^{-1}\mathrm{dp}$$

where *p* is allele frequency. Thus, retention of neutral alleles depends on both the effective and census effective population sizes, and there is no simple relationship between the number of alleles and either *N*_e_ or *N*_C_ (Figure 1).

## 3 RESPONSE TO SELECTION

The rate of change in response to natural selection is proportional to the amount of genetic variation present (Fisher, 1930). Heterozygosity is proportional to the amount of additive genetic variance at loci affecting phenotypic variation (Falconer & Mackay, 1996). Thus, heterozygosity is a good predictor of the potential of a population to evolve in the short term.

Low‐frequency alleles do not contribute much to the immediate response of a population to selection. Nevertheless, the limit of response to selection over many generations is determined by the initial allelic variation present (Caballero & García‐Dorado, 2013; James, 1971; Robertson, 1960).

For example, consider a gene that contributes to the flight endurance of a bird that has been held in captive propagation and is now going to be reintroduced into the wild. This locus in population A has two alleles at equal frequency that contribute 1 and 1.1 “endurance units,” respectively. Population B has these same two alleles at equal frequencies but it also has a third allele at a frequency of 0.01 that contributes 1.2 endurance units. A third population, C, has only the 1.0 endurance unit allele at a frequency of 0.99 and the 1.2 unit endurance allele at a frequency of 0.01.

What is the expected response to selection for increased flight endurance after these three populations are introduced into the wild? Populations A and B will show the greatest initial response because of their high heterozygosities. The presence of the high endurance allele in C will have little initial effect because this allele is initially at a low frequency. The magnitude of the long‐term response, however, is determined by the presence or absence of the high endurance allele. Thus, populations B and C will show a greater long‐term response to selection if the 1.2 unit endurance allele is not lost through genetic drift.

Both high heterozygosity and high allelic variation are desirable in populations being considered for reintroduction programs. Sufficient heterozygosity must be present to allow short‐term success of the species to survive in the wild. Allelic variation, however, is important for increasing the chances of long‐term survival.

## 4 EFFECT OF BOTTLENECKS

Bottlenecks have very different effects on the amount of heterozygosity and allelic variation in a population. A variety of tests have been developed to detect past bottlenecks in a population based on this phenomenon (see Peery et al., 2012).

The proportional loss of heterozygosity in a population per generation due to genetic drift is the following:

(3) $$\Delta H=-\frac{1}{2N_{e}}$$

The heterozygosity after *t* generations (*H*_t_) is the following where *H*_0_ is the initial heterozygosity (Crow & Kimura, 1970, page 102):

(4) $$H_{t}={\left(1-\frac{1}{2N_{e}}\right)}^{t}H_{0}$$

We use allelic diversity (*A*_D_) as a measure of the proportion of variation remaining at a locus after a bottleneck based on the number of alleles retained:

(5) $$A_{D}=\frac{A_{n}^{\prime }-1}{A_{n}-1}$$

where *A*_n_ is the initial number of alleles and *A*_n_′ is the number of alleles remaining after a bottleneck. This measure ranges from one, when all alleles are retained (*A*′_n_ = *A*_n_), to zero, when there is only one allele remaining (*A*′_n_‐1 = 0).

The effect of a bottleneck on the number of alleles present is more complicated than the effect on heterozygosity because it is dependent on both the number and frequencies of alleles present (Allendorf, 1986). The probability of an allele being lost in a single generation in an ideal population of size *N* is as follows:

(6) $${\left(1-p\right)}^{2N}$$

where *p* is the frequency of the allele. This is the probability of sampling all 2*N* gametes to create the next generation without selecting at least one copy of the allele in question. Rare alleles (*p* < 0.10) are especially susceptible to loss during a bottleneck.

In general, if an ideal population is reduced to *N* individuals for one generation then the expected total number of alleles (*A*′_n_) remaining at a locus is the following:

(7) $$E\left(A_{n}^{\prime }\right)=A_{n}-\sum_{j=1}^{A_{n}}{\left(1-p_{j}\right)}^{2N}$$

where *A*_n_ is the initial number of alleles and *p*_j_ is the frequency of the *j*th allele (Denniston, 1978).

Heterozygosity is relatively insensitive to the effects of bottlenecks. For example, a population experiencing a single‐generation bottleneck of two individuals is expected to lose only 25% (Equation 3) of its heterozygosity. Thus, 75% of the heterozygosity in a population will be retained even through such an extreme bottleneck. However, two individuals can only possess a maximum of four different alleles, regardless of the number of alleles segregating in the population.

**FIGURE 2:**
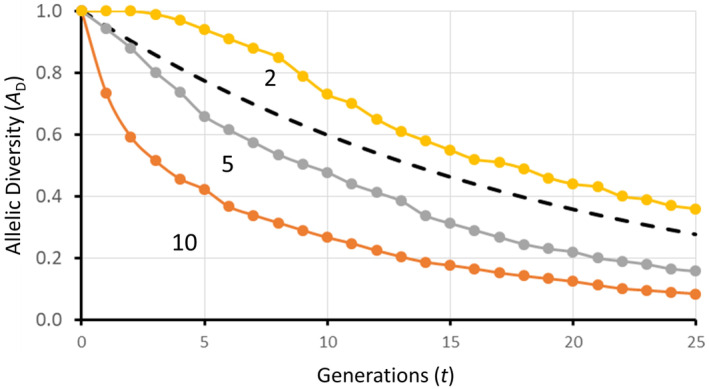
Simulation results (EASYPOP 2.0.1; Balloux, 2001) with 1000 repeats showing the mean allelic diversity (*A*_D_) remaining after *t* generations of a reduced population size of 10 (*N*_e_ = *N*_C_ = 10) beginning with 2, 5, and 10 equally frequent alleles. The numbers below each line indicate the number of equally frequent alleles initially present. The dashed line shows the heterozygosity expected to be retained using Equation (4).

The greater the number of alleles at a locus, the greater the proportion of allelic variation that is lost after a bottleneck. Figure 2 shows the simulation results of a bottleneck of 10 individuals in populations initially with 2, 5, and 10 equally frequent alleles.

## 5 HOW WELL DOES *N*_e_ DESCRIBE LOSS OF ALLELIC VARIATION?

A variety of corrections have been developed to describe the loss in heterozygosity that take into account different violations of the assumptions of the ideal population (e.g., unequal number of males and females, fluctuations in population size over time). Unfortunately, these corrections do not always accurately these assumptions of the ideal population on allelic variation.

### 5.1 Unequal number of males and females

The following formula (Crow & Kimura, 1970, page 108) provides an approximation of the effective population size with unequal numbers of females (*N*_f_) and males (*N*_m_):

(8) $$N_{e}\approx \frac{4N_{f}N_{m}}{N_{f}+N_{m}}$$

**FIGURE 3:**
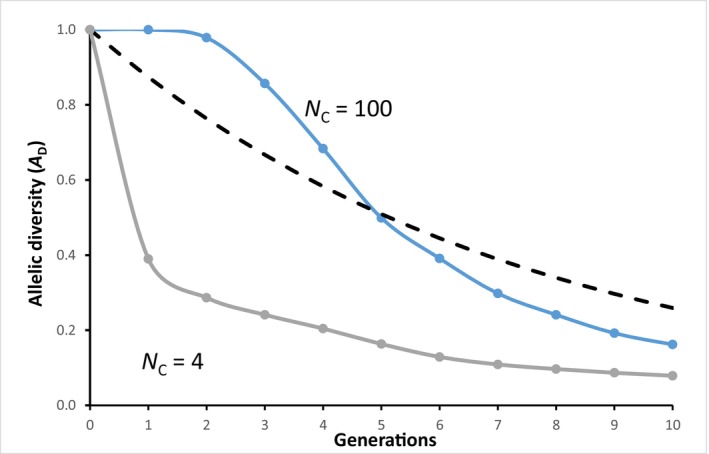
Simulation results (EASYPOP 2.0.1; Balloux, 2001) with 1000 repeats showing loss of allelic diversity in two populations with *N*_e_ = 4 having different values of *N*_C_. Each population initially had 10 alleles at equal frequency. The upper line is a population with 99 females and 1 male. The lower line is a population with 2 females and 2 males. The dashed line shows the heterozygosity expected to be retained in both populations using Equation (4).

Figure 3 shows the loss of allelic diversity in two cases where *N*_e_ = 4 using Equation 8 with different values of *N*_C_. As we saw before (Figure 1), populations with larger *N*_C_ maintain a greater number of alleles.

As an example, consider the hypothetical situation where a single tree is left standing after the clearcut of an entire population of ponderosa pine, a simultaneous hermaphrodite. This lone tree holds over 5000 seeds that were fertilized by pollen from a random sample of the entire pre‐clearcut population of 500 trees. What proportion of the heterozygosity and allelic variation present in the pre‐clearcut population would you expect to be present in a new population of 5000 trees founded from these seeds?

The effective population size in this population will be approximately 4 using Equation 8. Therefore, using Equation 3, we expect to lose approximately 1/2*N*_e_ = 12.5% of the heterozygosity from this population.

However, there is a good chance that almost all of the alleles are present in the 5000 seeds in which the paternally derived allele is a random sample of the 500 trees in the population. Using Equation 6, it is extremely unlikely to lose an allele at a frequency of 0.01 with a sample of 5000; (1–0.01)^5000^ is approximately zero. Therefore, this bottleneck will have no or little effect on allelic diversity.

### 5.2 Nonrandom number of progeny

This formula provides an approximation of the effective population size with nonrandom reproductive success (Crow & Kimura, 1970, page 349):

(9) $$N_{e}\approx \frac{4N_{C}-2}{2+V_{k}}$$

where *V*_k_ is variance of the number of offspring contributed to the next generation. A comprehensive treatment of this relationship is beyond the scope of this paper. Nevertheless, populations with large *N*_C_ and greater than random reproductive success would tend to maintain more alleles than populations having the same *N*_e_ but with small *N*_C_. We can see this in Figure 3 which is a special case of nonrandom number of progeny because of different numbers of males and females.

### 5.3 Fluctuations in population size

Natural populations sometimes fluctuate greatly in size. The rate of loss of heterozygosity (1/2 *N*) is proportional to the reciprocal of population size (1/*N*). Thus, generations with small population sizes will dominate the effect on loss of heterozygosity. This is analogous to the sex with the fewest individuals dominating the effect on loss of heterozygosity (Equation 8). Therefore, the average population size over many generations is a poor metric for the loss of heterozygosity over many generations.

We can estimate the effective population size over *t* generations by using the mean of the reciprocal of population size (1/*N*) in successive generations, rather than the mean of *N* itself. This is the harmonic mean (Crow & Kimura, 1970, page 110):

(10) $$N_{e}\approx \frac{t}{\sum \left(\frac{1}{N_{i}}\right)}$$

The duration of a bottleneck (intense versus diffuse) will affect heterozygosity and allelic diversity differently. Consider two populations that fluctuate in size over several generations with the same *N*_e_, and therefore the same loss of heterozygosity. A brief but very small bottleneck (intense) will cause substantial loss of allelic diversity. Thus, a diffuse bottleneck spread over several generations can result in the same loss of heterozygosity but will cause a much smaller reduction in allelic diversity.

England et al. (2003) subjected *Drosophila* populations to intense versus diffuse bottlenecks over 57 generations. The intense and diffuse bottlenecks were both designed to have an *N*_e_ of 100. The effects of these bottlenecks at nine microsatellite loci were evaluated. As expected, the intense and diffuse bottlenecks had similar effects on heterozygosity, but allelic variation was lower in the intense than the diffuse bottleneck treatments.

## 6 PRACTICAL APPLICATIONS

### 6.1 Loci with many alleles

The more alleles that are present at a locus, the greater the effect on *A*_D_ in comparison to *H* (Figure 2). Some loci that have many alleles are important for adaptation. These loci tend to be associated with disease resistance, mating type, or sex determination.

Major histocompatibility complex (MHC) loci are associated with disease resistance in many animal species (Hedrick, 2003). Loci that are part of the MHC system in vertebrates have been found to have many nearly equally frequent alleles. For example, Knafler et al. (2012) described allelic variation in 50 breeding pairs of the Magellanic penguins (*Spheniscus magellanicus*) at the MHC *DRß1* gene. They discovered 45 alleles in this sample of 200 gene copies. Two birds chosen at random from this population are expected to retain 75% of the heterozygosity (Equation 3). However, two birds can at best possess four of the 45 different *DRß1* alleles. Thus, at least 41 of the 45 alleles (91%) will be lost in a bottleneck of two individuals compared with the loss of just 25% of the heterozygosity.

R genes in plants also have many alleles. These loci act to detect pathogens and determine allele‐specific activation of defenses (Meyers et al., 2005). Reduced allelic variation in R genes has been shown to have harmful effects on seedling survival in small populations of tropical trees. Marden et al. (2017) found that smaller local tree populations with limited connectivity to other populations have reduced R gene diversity. This results in lower recognition‐dependent immune responses, along with greater susceptibility to species‐specific pathogens that may facilitate disease transmission in species with smaller local populations.

The self‐incompatibility locus (*S*) of many flowering plants generally has many alleles (Castric & Vekemans, 2004; Wright, 1965). In the simplest system, pollen grains can only fertilize plants that do not have the same *S* allele as carried by the pollen. New mutations will always have an advantage in this system so *S* loci generally have many alleles.

Emerson (1940) described 45 nearly equal frequency *S*‐alleles in the Organ Mountains evening primrose (*Oenothera organensi*), a narrow endemic plant that occurs in an area of ~50 km^2^ in the Organ Mountains, New Mexico, United States. Emerson originally thought that the total population size of this species was ~500 individuals. More recent surveys indicate that the total population size may be as great as 5000 individuals (Levin et al., 1979). Regardless of the actual population size, this is an enormous amount of variability at a single locus. As expected because of its small population size, this species has very little genetic variation at other loci as measured by protein electrophoresis (Levin et al., 1979).

Les et al. (1991) considered the demographic importance of maintaining a large number of *S*‐alleles in plant populations. A reduction in the number of *S*‐alleles because of small population size will reduce the frequency of compatible matings and may result in reduced seed set. Young and Pickup (2010) found that small populations (*N*_C_ < 100 plants) of the button wrinklewort (*Rutidosis leptorrhynchoides*) have low *S*‐allele diversity and mate availability and exhibit significant reductions in seed set relative to large populations (*N*_C_ > 1000 plants) with higher numbers of *S*‐alleles. Reinartz and Les (1994) concluded that some one‐third of the remaining 14 natural populations of the forked aster (*Aster furactus*) in Wisconsin had reduced seed sets because of a diminished number of *S*‐alleles.

Nearly 15% of animal species are haplodiploid in which sex is determined by genotypes at one or more hypervariable loci (ants, bees, wasps, thrips, whitefly, certain beetles, etc.; Crozier, 1971; Cook & Crozier, 1995). Heterozygotes at the sex‐determining locus are female, and the hemizygous haploids or homozygous diploid individuals are male (Packer & Owen, 2001). Most natural populations have been found to have 10–20 alleles at this locus. Therefore, loss of allelic variation caused by a population bottleneck will increase the number of diploid males produced by increasing homozygosity at the sex‐determining locus or loci.

### 6.2 Species in which *N*_C_ is much greater than *N*_e_

Hoban et al. (2020) reviewed published *N*_e_/*N*_C_ ratios in over 200 species. The only taxonomic group that was exceptional was marine fishes. They had a mean ratio of 0.024 in comparison to a mean of 0.354 in all 8 taxonomic groups. The median *N*_e_/*N*_C_ ratio in marine fishes was 2.67 × 10^−4^, in comparison to 0.303 in the other taxa. Hedgecock and Pudovkin (2011) reported similar low ratios in marine invertebrates.

Pinsky and Palumbi (2014) reported that overfished populations have approximately 2% lower heterozygosity and 12% lower allelic richness than populations that are not overfished. They also performed simulations that suggest that their estimates likely underestimate the actual loss of rare alleles by a factor of three or four. Ryman et al. (1995) argued that the loss of alleles because of overfishing may actually be more dramatic in large populations than in small ones.

For example, there is evidence of severe overfishing in the Atlantic herring (*Clupea harengus*) in the Baltic Sea (Baltic herring). Census population size in the central Baltic Sea dropped from about 31 to 9 billion in the period 1974–2021 (table 4.17, International Council for the Exploration of the Sea (ICES), 2021). Under mutation‐drift equilibrium, with a mutation rate of *μ* = 10^−7^, and a ratio of effective to census size of about 0.024 for marine fishes (Hoban et al., 2020), the expected change of heterozygosity would be negligible from 0.997 to 0.989 (Equation 1). In contrast, the effect on the expected number of alleles would be much more dramatic going from about 5500 down to 1700, a loss of about 70% (Equation 2). Thus, although heterozygosity essentially would remain unchanged, most of the alleles have been lost.

**FIGURE 4:**
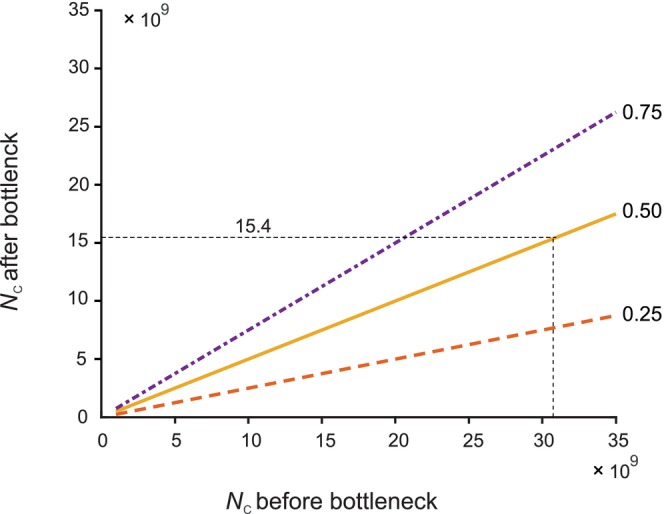
Bottleneck sizes that would retain a given proportion (25%, 50%, or 75%) of alleles. For example, the census size was *N*_C_ = 30.7 billion before the bottleneck in the Baltic herring. Retention of 50% of the alleles would require a minimum *N*_C_ = 15.4 billion after the bottleneck. The trajectories are constructed using *N*_e_/*N*_C_ = 0.024 and *μ* = 10^−7^, assuming migration‐drift equilibrium before and after the bottleneck. The curves can be used for evaluating the expected effect of any change of *N*_C_ on allelic reduction as long as *N*_e_/*N*_C_ remains the same over the bottleneck, and *N*_e_ *μ* is not too small (*N*_e_ *μ* > 1). See text for details.

The minimum census size of the Baltic herring required to retain 25%, 50%, and 75% of the original expected number of alleles after a bottleneck is shown in Figure 4. For example, a census size of 15.4 billion would have been necessary to maintain half of the original expected number of alleles.

It is important to note that the trajectories in Figure 4 are straight lines. Thus, there is a linear relationship between the proportional reduction of *N*_C_ and the expected number of alleles at the new equilibrium after the bottleneck. In spite of the intricate calculations required to evaluate Equation 2, the proportional loss of alleles can be obtained directly from the reduction of *N*_C_ as long the ratio of effective to census size (*N*_e_/*N*_C_) is the same before and after the bottleneck. For example, a 42% reduction in *N*_C_ will result in a 42% reduction in the expected number of alleles, regardless of the initial value of *N*_C_ as long as *N*_e_/*N*_C_ has not changed. We have verified this relationship by numerical evaluation of Equation 2. Some minor approximations are needed, but results are reliable as long as *N*_e_ *μ* after the bottleneck is not too small (say *N*_e_ *μ* > 1).

This analysis assumes drift‐mutation equilibrium, which is unlikely to be reached. The number of generations required to reach drift‐mutation equilibrium is on the order of the reciprocal of the mutation rate (Nei et al., 1975). This would be approximately 10 million generations in this example with *μ* = 10^−7^. Therefore, the loss of alleles following population declines that occurred recently is likely to be less than presented here.

### 6.3 Metapopulations: Fragmentation and gene flow

Hill et al. (2023) have considered the effects of fragmentation and gene flow on both heterozygosity and allelic variation. They conclude that episodic fragmentation and gene flow have contrasting effects on heterozygosity and allelic variation. Fragmentation into many, small subpopulations with periods of infrequent gene flow, preserves allelic variation at the expense of heterozygosity. In contrast, fragmentation into a few, large populations maintains heterozygosity at the expense of allelic variation. The strength of the trade‐off between heterozygosity and allelic variation depends on the amount of gene flow and the frequency of gene flow events (Caballero & García‐Dorado, 2013).

### 6.4 Genetic monitoring

Genetic monitoring programs have become an essential aspect of genetic management for many species. Such programs can provide a powerful means to detect loss of genetic variation. Monitoring of allelic variation is much more difficult than monitoring heterozygosity. Monitoring the number of alleles requires large samples to detect the majority of alleles, many of which are expected to occur in low frequencies.

In addition, allelic variation is generally not estimated with sequence data. Single nucleotide polymorphisms (SNPs) generally have just two alleles and have limited power in comparison with highly polymorphic loci that often have many alleles (e.g., microsatellites). However, multiple SNPs that occur within the same region can be used to derive multi‐allelic markers (Kidd et al., 2014; Leitwein et al., 2019). Developing methods to estimate allelic variation using DNA sequence data would be extremely helpful to compare the amount of allelic variation in conspecific populations. One could take a series of say 1000 base pair segments and count the number of haplotypes present. The number of haplotypes present in different populations would provide a useful analogue of allelic variation to compare populations.

## 7 RECOMMENDATIONS

Effective population size is the basis for the 50/500 guideline of Franklin (1980) that has become widely applied in conservation biology (Hoban et al., 2020). This rule suggests that for an isolated population, *N*_e_ > 50 is needed to avoid the harmful effects of inbreeding depression in the short‐term. And in the long‐term, *N*_e_ > 500 is needed to maintain sufficient genetic variation to allow adaptation to environmental changes (Jamieson & Allendorf, 2012).

As shown in this paper, predicting the rate of loss of allelic variation is much more complicated than predicting the rate of loss of heterozygosity. The rate of loss of allelic variation depends upon the frequency of alleles at a locus, and it will therefore be different for every locus. Therefore, there are no simple recommendations similar to the 50/500 guideline that we can propose to provide guidance for maintaining allelic variation.

There is general agreement between the rate of loss of heterozygosity and allelic variation. Nevertheless, there are some conflicts between rate of loss of heterozygosity and allelic variation when using *N*_e_ alone to make decisions. For example, consider establishing a conservation hatchery population of fish from an endangered wild population. In some cases, many more males than females may be available. Should we make an effort for each of these males to contribute to our founding population? The answer would be “no” if using effective population size as the sole criterion because additional males will have little effect on effective population size (Equation 8). Nevertheless, the genetic contribution of such additional males would increase the total number of alleles present in our founding population.

Perhaps the greatest danger associated with the use of only heterozygosity to predict the loss of genetic variation is the overly optimistic estimate of the effects of bottlenecks. Little heterozygosity is expected to be lost through even severe bottlenecks of short duration. Nevertheless, such bottlenecks are expected to have a major effect on allelic variation and may have a significant effect on long‐term adaptability and survival. Therefore, populations that have experienced a severe but brief bottleneck are more vulnerable in the long term than populations that have gone through an extended but mild bottleneck. In addition, large populations that have undergone a decline might have lost substantial allelic variation even if the current population size is very large.

Our primary recommendation is that those responsible for making conservation decisions be aware of the limitations of *N*_e_ to predict the rate of loss of allelic variation. The possible loss of allelic variation should be taken into account for evaluating the conservation status of species as well as effective population size.

## CONFLICT OF INTEREST STATEMENT

The authors declare no conflict of interest.

## Data Availability

This study generated no new data except by simulation using EASYPOP 2.0.1 (Balloux, 2001).
